# Strong Lateral Mode Confinement by Embedding SiO_2_ Nanospheres in the DBRs of GaN-Based VCSELs

**DOI:** 10.3390/mi17050544

**Published:** 2026-04-29

**Authors:** Huanqing Chen, Menglai Lei, Linghai Meng, Zihao Chu, Weihua Chen, Xiaodong Hu

**Affiliations:** 1 State Key Laboratory of Artificial Microstructure and Mesoscopic Physics, School of Physics, Peking University, Beijing 100871, China; leimenglai@pku.edu.cn (M.L.); 1901110148@pku.edu.cn (Z.C.); chenwh@pku.edu.cn (W.C.); 2 Guangxi Hurricane Chip Technology Co., Ltd., Guangxi 545003, China; 15910329586@163.com

**Keywords:** GaN-based VCSEL, curved DBR, SiO_2_ nanospheres, lateral confinement

## Abstract

In this work, we report the realization of curved distributed Bragg reflectors (DBRs) without the need for lithography to achieve strong lateral confinement in a GaN-based vertical cavity. By embedding SiO_2_ nanospheres during deposition, curved DBRs with a funnel-shaped cross-section were fabricated. Based on the formed curved DBRs, a vertical cavity with a quality factor exceeding 2800 and a mode volume below 0.14 μm^3^ was successfully fabricated. The optical pumping threshold power of a vertical cavity surface-emitting laser (VCSEL) with a curved DBR was reduced to 76 nW, which is one order of magnitude lower than that of the same VCSEL with double-planar DBRs. Near-field patterns revealed that the curved-DBR VCSEL emits a circularly symmetric TEM_00_ mode with a full width at half maximum (FWHM) of only 1.8 μm. We believe this is an effective technique for fabricating low-threshold or small-aperture VCSELs.

## 1. Introduction

Since their invention in the 1980s, VCSELs have found widespread application in optical communications, optical sensing, and consumer electronics due to their low threshold and circular optical characteristics [1,2]. Compared to conventional edge-emitting lasers, VCSELs feature smaller volumes and more pronounced microcavity effects [3]. Among various material platforms, GaN-based VCSELs are particularly favored for their ability to cover the ultraviolet to green spectral range [4]. However, the performance of GaN-based VCSELs has long been constrained by challenges in achieving efficient lateral optical confinement, which is critical for reducing threshold power and supporting single-mode operation [5].

In conventional GaAs-based VCSELs, lateral confinement is typically achieved via selective oxidation of AlGaAs layers, forming an oxide aperture that provides both current and optical confinement [6,7]. Unfortunately, due to the lack of suitable oxidation processes, this method cannot be directly applied to GaN-based devices. Consequently, researchers have explored alternative strategies, such as employing etched mesa or buried dielectric structures to create refractive index gradients [8]. For instance, Kuramoto et al. demonstrated a buried mesa structure that effectively reduced internal loss by over 68% in GaN-based VCSELs [9]. Nevertheless, these methods often introduce abrupt changes in refractive index, leading to scattering losses and a decrease in the cavity quality factor (Q), particularly in devices with small apertures [10]. Ding et al. investigated the effects of embedded defect shapes on micro cavity quality factor (Q) and mode volume, and they found that cylindrical mesas with steep sidewalls cause severe scattering, limiting Q to below 1000 [11]. In contrast, defects with smooth shapes can reduce the mode volume while maintaining a microcavity Q-factor above 20,000.

To address these issues, some advanced fabrication techniques such as gray-scale lithography and thermal reflow have been proposed to create curved microcavities [12,13]. However, these methods are usually complex and costly. In this paper, we propose a simple and efficient lithography-free method to fabricate curved DBRs on the micrometer scale. This technique utilizes the burying effect of SiO_2_ nanospheres during DBR coating to form a funnel-shaped DBR with smooth sidewalls, providing strong lateral confinement without compromising cavity quality. The resulting VCSELs exhibit significantly reduced threshold power, high Q-factor, and fundamental transverse mode operation, offering a promising pathway toward compact, low-power GaN-based coherent light sources.

## 2. Experiment

**Figure 1:**
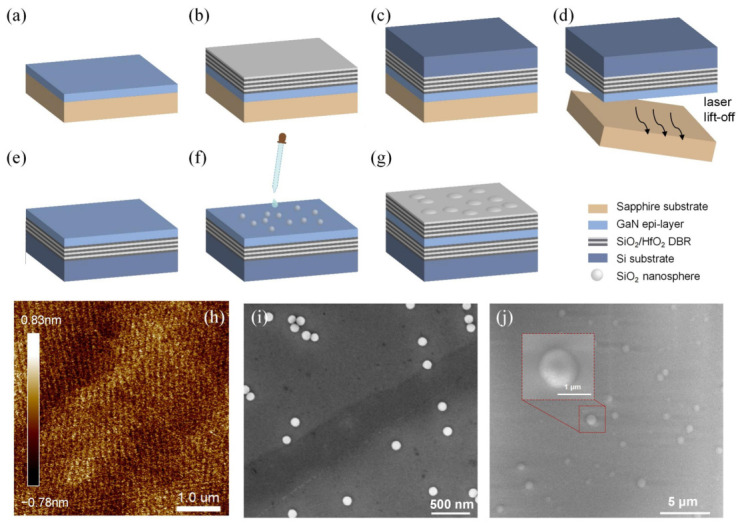
(**a**–**g**) Schematic diagram of the VCSELs with curved DBRs. (**h**) AFM image of the GaN backside after CMP. (**i**) SEM image of SiO_2_ nanospheres coated on the surface. (**j**) SEM image of the curved DBR formed by embedding SiO_2_ nanospheres.

Figure 1a–g illustrate the fabrication process for fabricating GaN-based VCSELs with curved DBRs in this paper. First, 12 pairs of InGaN/GaN MQW were grown on a c-plane sapphire substrate via metal–organic chemical vapor deposition (MOCVD); the MQW period was designed to be ~83 nm to form resonant periodic gain (RPG) matched to the cavity optical field [14,15]. Details on the RPG growth parameters can be found in our previous work [16]. The PL wavelength of the active region is ~425 nm, with a surface roughness of 0.32 nm. Subsequently, 15 pairs of HfO_2_/SiO_2_ were deposited onto the surface by electron beam evaporation to attain a reflectivity exceeding 99%. The sapphire substrate was then removed using a conventional process flow that included wafer bonding, laser lift-off, and chemical mechanical polishing (CMP), thereby exposing the bottom surface of the active region [17,18]. As shown in Figure 1h, the backside surface roughness after CMP was reduced to 0.31 nm, meeting the requirements for high-quality optical cavities.

To fabricate the curved DBRs, a diluted solution containing SiO_2_ nanospheres was spin-coated onto the sample surface and baked at 150 °C to evaporate the solvent (see Figure 1f). Figure 1i shows the scanning electron microscopy (SEM) image of dispersed SiO_2_ nanospheres; most SiO_2_ nanospheres exhibit low surface density distribution (density = 1 × 10^7^~1 × 10^8^ cm^−2^) and non-contacting arrangement, making them suitable for fabricating isolated curved DBRs. Although the distribution of SiO_2_ nanospheres is currently rather random, it will be possible in the future to prepare array structures using a template-assisted method or other techniques [19]. The inset reveals that the spheres have diameters of ~90 nm with relatively smooth surfaces. Finally, 15 pairs of HfO_2_/SiO_2_ were deposited onto the sample surface to embed the SiO_2_ nanospheres, completing the microcavity fabrication. Figure 1j provides a top-view SEM image of the top DBR surface, showing uniformly distributed dome-like structures with circular bases. The magnified inset reveals that the curved DBRs have diameters of ~1 µm.

**Figure 2:**
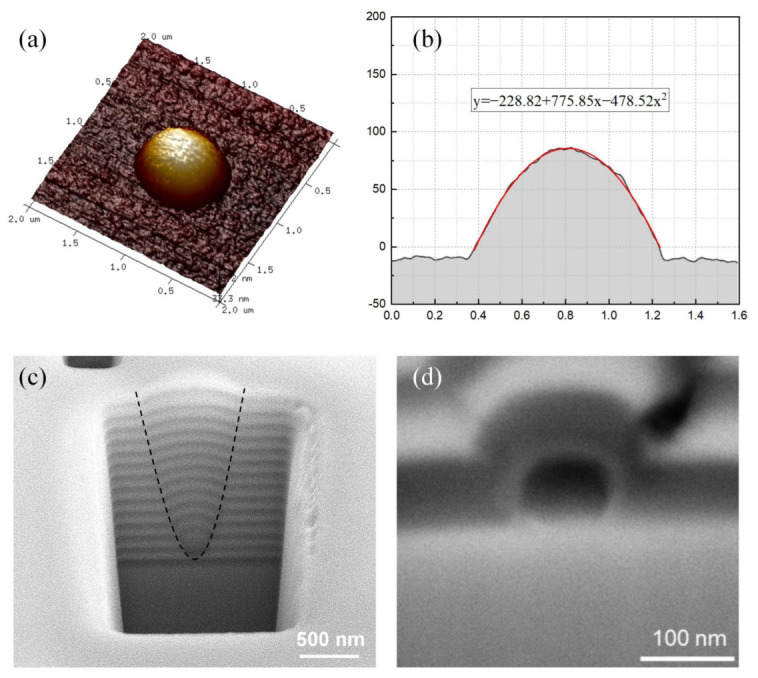
(**a**) AFM image of the curved DBR. (**b**) Central cross-section fitted with a polynomial. (**c**) SEM image of the curved DBR cross-section. (**d**) Enlarged SEM image of the curved DBR base.

The surface profile of the curved DBR was characterized by atomic force microscopy (AFM). As shown in Figure 2a, the AFM image reveals a smooth, dome-shaped protrusion with a maximum height of ~92 nm relative to the planar substrate surface. Figure 2b shows the cross-section profile extracted from the center of Figure 2a. The profile was fitted by a second-order polynomial expression: y = −0.228 + 0.775x + 0.478x^2^, giving a central radius of curvature R = 1.04 μm. To gain deeper insight into the formation mechanism of the curved DBR, the evolution of its structure during the deposition process was investigated using focused ion beam (FIB) milling combined with SEM. Figure 2c shows the cross-section SEM image of a curved DBR tilted at 52°. It is clearly shown that the curved DBR structure originates from the sample surface and expands radially outward during deposition, with its diameter increasing progressively from 0 μm at the base to approximately 0.85 μm at the top surface. This gradual expansion is characteristic of isotropic deposition processes, where material is deposited uniformly in all directions from a seed point. Figure 2d presents an enlarged SEM image of the interface between the curved DBR and GaN, revealing that the protrusion is initiated by a buried SiO_2_ nanosphere. The nanosphere acts as a localized seed, perturbing the otherwise planar deposition front and giving rise to the curved profile.

Interestingly, the boundary of curved DBRs exhibits a parabolic-like contour. On a spherical seed, isotropic deposition proceeds outward in concentric fronts because the velocity of the isotropic deposition front is perpendicular to the original substrate plane and of equal magnitude [20]. Theoretical modeling based on the level set method predicts that such isotropic growth conditions lead to the formation of strictly parabolic boundary profiles. And interestingly, the curvature and focal point of the resulting parabola are governed by the isotropic deposition ratio, defined as D_iso_/D_sum_ (see Supporting Information), where D_iso_ represents the isotropic component of the deposition rate and D_sum_ is the total deposition rate [21,22].

## 3. Results and Discussion

Optical pumping of the curved VCSEL and adjacent planar VCSEL regions was performed using a confocal micro-PL system, which enables precise spatial selection and high-resolution spectral acquisition [23]. The pump source was a nanosecond pulsed laser (wavelength 355 nm, pulse width 8.0 ns, frequency 1.0 kHz), which was focused by the objective microscope lens to a 1 µm spot size. The power of the pump source mentioned in this paper was measured using a power meter, which calculates the average power obtained by integrating the energy of multiple consecutive pulses. All measurements were conducted under identical excitation power and ambient conditions to eliminate external variables and isolate the effect of cavity geometry on lasing behavior.

**Figure 3:**
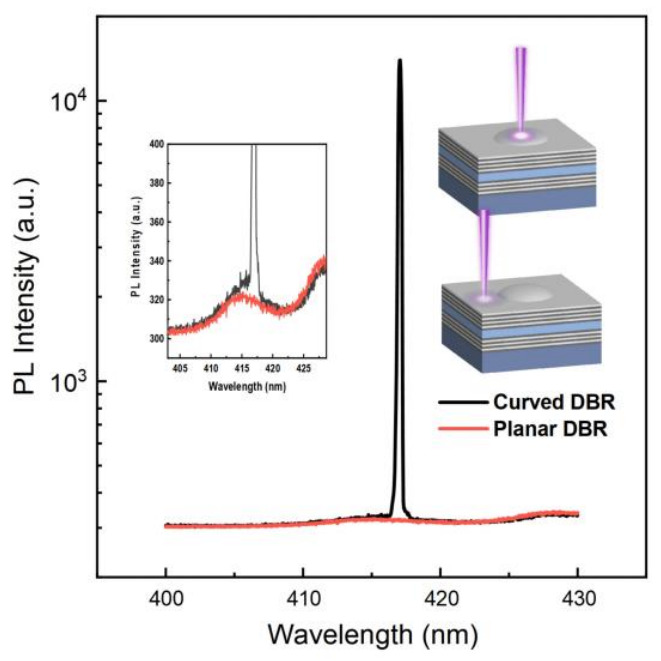
PL spectra of curved DBR and planar DBR regions excited with identical pump power.

Figure 3 compares the PL spectra of the corresponding regions under identical excitation power. The curved VCSEL shows significantly higher PL intensity and spectral narrowing, which is strong evidence of lasing. The sharp resonance peak emerges at a wavelength consistent with the cavity mode, indicating efficient coupling of the gain medium to the optical mode supported by the curved DBR structure. However, the PL spectrum from the planar VCSEL remains a broad spontaneous emission envelope without significant cavity enhancement. Notably, the spontaneous emission spectra of both structures are nearly identical. This indicates that the active region material properties are consistent across the sample. The preservation of identical spontaneous emission characteristics also eliminates any unintended variations in gain medium quality or excitation efficiency, thereby strengthening the conclusion that the curved DBR is responsible for the enhanced optical confinement and reduced threshold.

**Figure 4:**
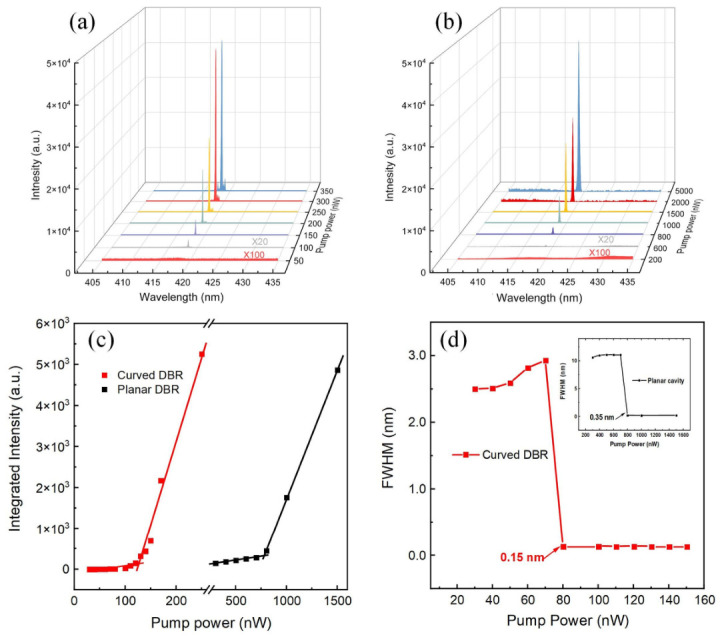
PL spectra of (**a**) curved VCSEL and (**b**) planar VCSEL at different excitation powers. (**c**) Integrated PL intensity and (**d**) spectrum FWHM as a function of excitation powers for curved VCSEL and planar VCSEL.

To further investigate the lasing characteristics and extract key performance parameters of the two VCSEL structures, power-dependent PL spectra were measured, as shown in Figure 4a,b. Both devices exhibit a distinct transition from spontaneous emission to stimulated emission, characterized by a sudden decrease in the FWHM and a sharp increase in the integrated PL intensity. However, the curved VCSEL demonstrates a significantly lower threshold compared to its planar counterpart.

The integrated intensity of curved and planar VCSELs is plotted as a function of pump power to extract the lasing threshold. It can be seen in Figure 4c that both VCSELs exhibit superlinear intensity growth. The threshold power for the curved VCSEL is determined to be 76 nW (0.99 mJ/cm^2^), while the planar VCSEL exhibits a threshold power of 820 nW (10.5 mJ/cm^2^), which indicates that the curved DBR structure reduces the VCSEL threshold by nearly an order of magnitude. This substantial reduction in threshold power provides compelling evidence that the curved DBR structure significantly enhances the optical confinement and reduces the modal gain required to achieve lasing. The observed threshold reduction can be attributed to several interrelated factors. First, the curved DBR effectively reduces the mode volume (V) of the cavity, as confirmed by finite-difference time-domain (FDTD) simulations presented later in this work [24]. According to the Purcell effect, a smaller mode volume enhances the spontaneous emission coupling efficiency (β) into the lasing mode, thereby lowering the threshold power [25,26]. Second, the smooth parabolic profile of the curved DBR minimizes scattering losses at the cavity boundaries, preserving a high quality factor and enabling more efficient energy storage within the active region [27]. Third, the curved geometry provides lateral confinement that focuses the optical field into the gain region, increasing the overlap between the optical mode and the InGaN/GaN multiple quantum wells [28].

Another crucial metric for the optical cavity is the quality factor (Q-factor), extractable from the spectral linewidth [29]. The Q-factor can be extracted from the spectral linewidth above threshold using the relation Q = λ/Δλ, where λ is the resonant wavelength and Δλ is the FWHM of the lasing peak. As shown in Figure 4d, the spectral linewidth of the planar VCSEL drops to 0.35 nm, corresponding to a Q-factor of ~1200. In contrast, the curved VCSEL exhibits a linewidth of only 0.15 nm (Q-factor = 2800) above the threshold, which is over 50% higher than the planar VCSEL. The Q-factor of the curved VCSEL is at an upper-intermediate level among previously reported GaN-based VCSELs with a planar and embedded structure [30,31,32,33]. This substantial enhancement in Q-factor directly reflects the reduced optical losses in the curved cavity, consistent with theoretical predictions for microcavities with smooth, curved boundaries. The simultaneous improvement in both Q-factor and threshold observed here, therefore, provides convincing evidence for the advantages of the curved DBR design, which demonstrates that the SiO_2_ nanosphere embedding method not only simplifies fabrication but also yields cavities with optical quality approaching that of more complex lithographically defined structures.

**Figure 5:**
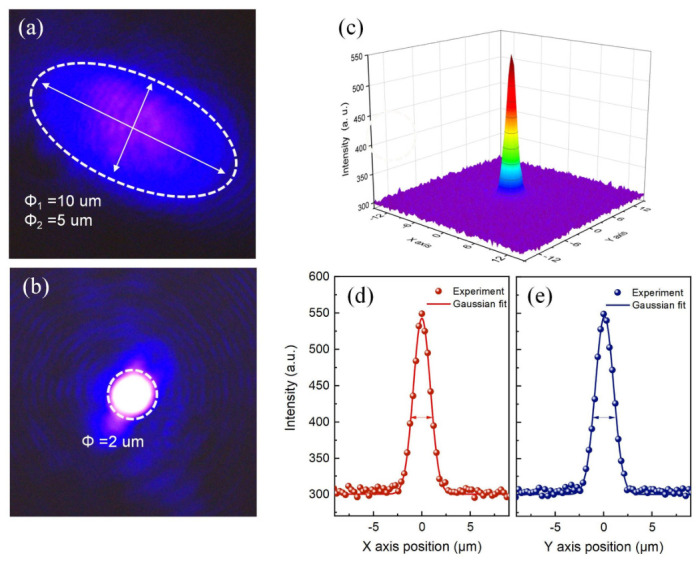
Near-field patterns of (**a**) planar VCSELs and (**b**) curved VCSELs. (**c**) Intensity distribution of the emission from curved VCSELs. (**d**,**e**) Gaussian fits along the *x*- and *y*-axes.

To directly visualize the spatial characteristics of the emission and assess the lateral confinement capability of the two cavity designs, near-field patterns of the planar and curved VCSELs were captured above the lasing threshold using a charge-coupled device (CCD) camera integrated with the micro-PL system. Figure 5a,b shows the near-field pattern of planar and curved VCSELs above their respective threshold. Both structures exhibit stable, well-defined emission spots surrounded by distinct interference fringes, confirming good spatial coherence and the establishment of a coherent optical mode within the cavity [34].

The shape of the planar VCSEL spot is roughly elliptical with major and minor axes of ~10 μm and 5 μm. This irregular and asymmetric mode profile can be attributed to the absence of engineered lateral optical confinement in the planar structure. In such devices, the transverse mode profile is largely determined by unintentional variations in the active region, such as local fluctuations in quantum well thickness, composition, or strain distribution, which can create weak index guiding or gain guiding effects [35]. It is important to emphasize that this elliptical shape is not an artifact of the excitation geometry, as the pump laser spot size was carefully maintained at 1 μm, which is smaller than the observed emission area. Nevertheless, the curved VCSEL exhibits a significantly brighter and more compact emission spot with a distinctly circular symmetric distribution. The enhanced brightness is consistent with the higher Q-factor and lower threshold observed in the power-dependent measurements, indicating more efficient extraction of light from the cavity. The circular symmetry of the spot provides convincing evidence of effective lateral optical confinement imposed by the curved DBR structure. Unlike the planar cavity, where mode formation is uncontrolled, the curved DBR acts as a waveguide that defines a well-confined optical aperture, forcing the mode to adopt a symmetric profile aligned with the curvature of the DBR.

To quantitatively analyze the mode profile, the intensity distribution of the curved VCSEL emission was extracted along orthogonal axes from the CCD image, as shown in Figure 5c [36]. Both the *x*-axis and *y*-axis intensity distributions follow standard Gaussian profiles, with FWHM values of 1.76 μm and 1.85 μm, as illustrated in Figure 5d,e. The spot size aligns closely with the shape of the curved DBR, with the mode extending slightly beyond the physical aperture, which is expected due to the evanescent field penetration into the DBR mirrors. This confirms that the curved DBR effectively confines the light field beneath it, thereby generating a high-quality Gaussian beam with the fundamental TEM_00_ mode.

The electric field distribution in the curved VCSEL was simulated using the 3D finite-difference time-domain (FDTD) method. The simulation domain was constructed based on the actual device geometry, with both the top and bottom distributed Bragg reflectors (DBRs) consisting of 15 pairs of HfO_2_/SiO_2_ dielectric layers, designed for a central wavelength of 420 nm. The refractive indices of HfO_2_ and SiO_2_ were taken as 2.05 and 1.48, respectively, at the operating wavelength. The funnel-shaped curved DBR structure was modeled according to the cross-sectional SEM image, ensuring that the simulated geometry accurately reflects the fabricated device morphology. Perfectly matched layer (PML) boundary conditions were applied to all simulation boundaries to absorb outgoing waves and prevent spurious reflections.

**Figure 6:**
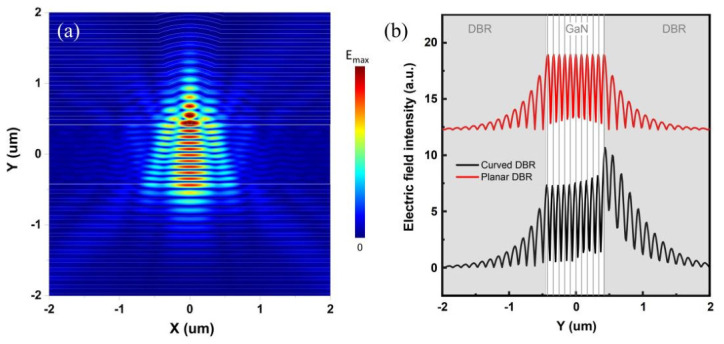
(**a**) 3D FDTD simulation of the electric field distribution in the curved VCSEL. (**b**) Electric field comparison between curved and planar VCSELs at x = 0.

Figure 6a shows the electric field intensity distribution of the curved VCSEL at its resonant wavelength of ~420 nm. It can be observed that the electric field mode within the active region is primarily concentrated beneath the curved DBR, and rapidly decays in the lateral directions outside the curved region. This observation provides direct evidence that the curved DBR functions as an effective lateral optical confinement structure, guiding the optical mode and preventing it from spreading into the unpumped planar regions.

The mode volume, which reflects the spatial confinement of photons, is defined as [37]:

(1) $$V=\frac{\int_{V_{in}}\varepsilon_{r}\left(r\right){\left|E\left(r\right)\right|}^{2}d^{3}r}{\max\left(\varepsilon_{r}\left(r\right){\left|E\left(r\right)\right|}^{2}\right)}$$

where V is the cavity volume, E(r) is the electric field strength, and ε_r_ is the relative permittivity. According to Equation (1), the mode volume of the curved VCSEL is calculated to be only 0.14 μm^3^. Figure 6b illustrates the electric field intensity distribution at x = 0 for both the curved and planar VCSELs. Notably, the maximum intensity of the curved VCSEL occurs within the SiO_2_ nanosphere. This phenomenon can be explained by the focusing effect of the surrounding concave DBR. The SiO_2_ nanospheres are conformally embedded within a curved DBR mirror stack, positioning them at the center of curvature of the surrounding curved DBR. This arrangement is analogous to a spherical lens in a confocal cavity, capable of focusing light onto the center of the nanospheres [38,39,40]. Furthermore, embedding SiO_2_ nanospheres in a curved microcavity creates a central region with a longer effective cavity length; thus, the effective refractive index of the central region is higher than the surrounding areas, generating strong localization effects and reducing the modal volume [41].

## 4. Conclusions

In conclusion, we have demonstrated a simple and scalable lithography-free method for fabricating curved DBRs in GaN-based VCSELs by embedding SiO_2_ nanospheres during dielectric deposition. The resulting funnel-shaped DBRs provide smooth, parabolic-like sidewalls that enable strong lateral optical confinement without compromising cavity quality. The fabricated curved VCSELs exhibit a high Q-factor exceeding 2800, a mode volume as low as 0.14 μm^3^, and a tenfold reduction in lasing threshold compared to planar cavities. Near-field pattern confirms stable, single-mode operation with a circular Gaussian profile, highlighting the effectiveness of the curved DBR in mode selection and confinement. We believe this method can be readily extended to other material systems and device architectures, paving the way for compact, low-threshold coherent light sources for applications in integrated photonics and quantum sensing.

## Data Availability

The original contributions presented in this study are included in the article/Supplementary Materials. Further inquiries can be directed to the corresponding authors.

## Supplementary Materials

The following supporting information can be downloaded at: https://www.mdpi.com/article/10.3390/mi17050544/s1. Figure S1. (a) PL Emission Spectrum of the InGaN/GaN MQWs, (b) Omega-2theta XRD curve of the InGaN/GaN MQWs on (002) plane, (c) The (004) and (-105) reciprocal space mapping (RSM) of the InGaN/GaN MQWs. Figure S2. The shapes of deposited DBRs on (a) rectangular, (b) trapezoidal, (c) Gaussian, (d) triangular, and (e) circular protrusions simulated using the level set model. (f) Boundary morphology of curved DBRs at different isotropic deposition ratio (F = Diso/Dsum). Figure S3. Simulated light field distribution in (a) planar and (b) curved VCSELs, (c) Light field distribution extracted along y = 0 in curved VCSELs. Figure S4. (a) SEM image of a coupled curved VCSELs pair. (b) Near-field patterns of the coupled VCSELs when pump spot is aligned with the central region and (c) one of the curved DBRs. (d) Emission spectra of the coupled VCSELs in two pumping configurations. Table S1. recent Q-factor reports on GaN-based VCSEL. References [42,43] are cited in the Supplementary Materials.
