# Supplementary material for: Strong Lateral Mode Confinement by Embedding SiO2 Nanospheres in the DBRs of GaN-Based VCSELs
Source: Micromachines (Basel). 2026 Apr 29;17(5):544. doi: 10.3390/mi17050544 (PMC13208928; doi:10.3390/mi17050544)
Supplement: Supplementary file 1 [file micromachines-17-00544-s001.zip › micromachines-4225790-supplementary.pdf]

Supplementary Materials

Strong Lateral Mode Confinement by Embedding SiO_2 Nanospheres in the DBRs of GaN-Based VCSELs

Huanqing Chen [1,*], Menglai Lei [1], Linghai Meng [2], Zihao Chu [1], Weihua Chen [1] and Xiaodong Hu [1,*]

[1] State Key Laboratory of Artificial Microstructure and Mesoscopic Physics, School of Physics, Peking University, Beijing 100871, China; leimenglai@pku.edu.cn (M.L.); 1901110148@pku.edu.cn (Z.C.); chenwh@pku.edu.cn (W.C.)
[2] Guangxi Hurricane Chip Technology Co., Ltd., Guangxi 545003, China; 15910329586@163.com
* Correspondence: chq1998@pku.edu.cn (H.C.); huxd@pku.edu.cn (X.H.)

1. InGaN/GaN MQWs with a Resonant Periodic Gain (RPG) Structure

As shown in Figure S1(a), the PL emission wavelength of the MQW is centered around 426 nm. The satellite peaks in the XRD curve indicates sharp interfaces within the MQW, with a period of approximately 82 nm. The RSM images of (004) and (-105) plane reveal that the InGaN layer shares the same in-plane lattice constant as the GaN layer.

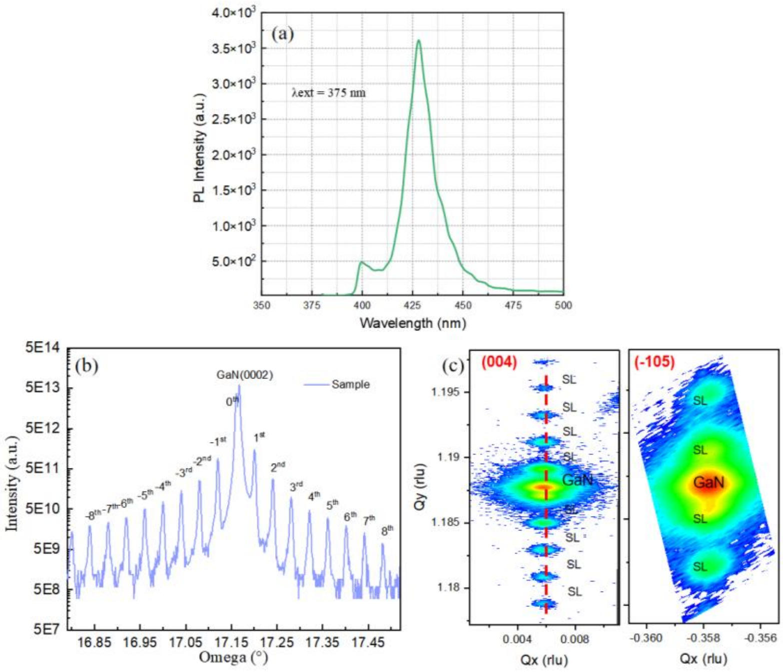

Figure S1. (**a**) PL Emission Spectrum of the InGaN/GaN MQWs, (**b**) Omega-2theta XRD curve of the InGaN/GaN MQWs on (002) plane, (**c**) The (004) and (-105) reciprocal space mapping (RSM) of the InGaN/GaN MQWs.

Academic Editor: Wei Liu

Received: 12 March 2026
Revised: 14 April 2026
Accepted: 16 April 2026
Published: 19 April 2026

Copyright: © 2026 by the authors. Submitted for possible open access publication under the terms and conditions of the Creative Commons Attribution (CC BY) license.

2. Shape Evolution of the Curved DBRs

As shown in Figure S2(a~e), the level set model was employed to simulate shape of deposited DBR under different surface geometries. Based on geometric principles, it can be proven that the DBR interface deposited on circular protrusion is strictly parabolic, and the focus of the parabola is determined by the isotropic deposition ratio (Diso/Dsum).

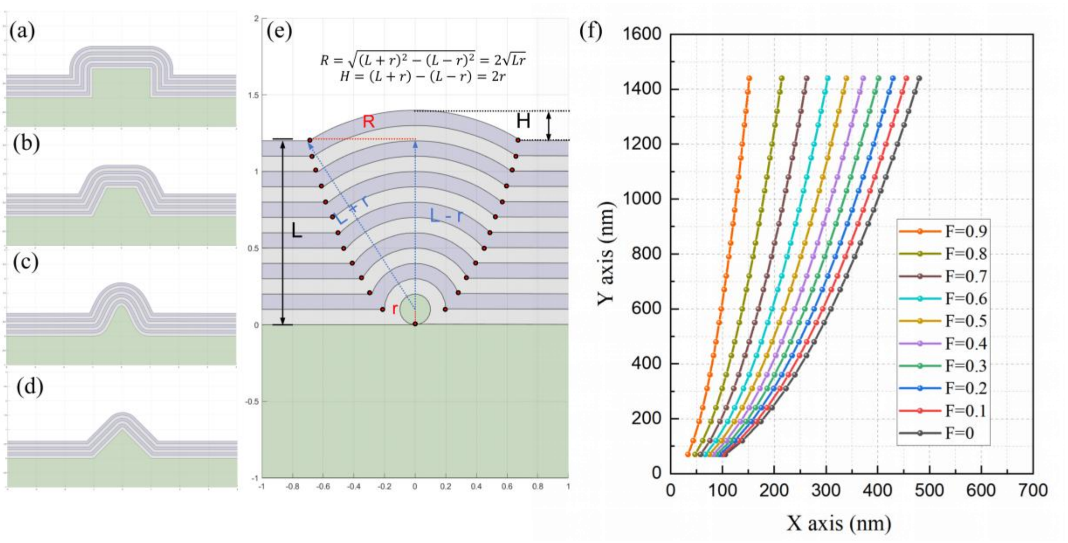

Figure S2. The shapes of deposited DBRs on (**a**) rectangular, (**b**) trapezoidal, (**c**) Gaussian, (**d**) triangular, and (**e**) circular protrusions simulated using the level set model. (**f**) Boundary morphology of curved DBRs at different isotropic deposition ratio (F = D_{iso}/D_{sum}).

3. FDTD Simulation Results

As shown in Figure S3 (a), the light field inside the planar cavity is uniformly distributed in-plane, since there is no lateral confinement structures. Figure S3 (c) showed the light field distribution inside the curved VCSEL in the horizontal direction. The electric field intensity is primarily concentrated in the central region with a half-width of approximately 1 μm, which is very close to the 1.76 μm beam waist size of the incident spot observed in experiments.

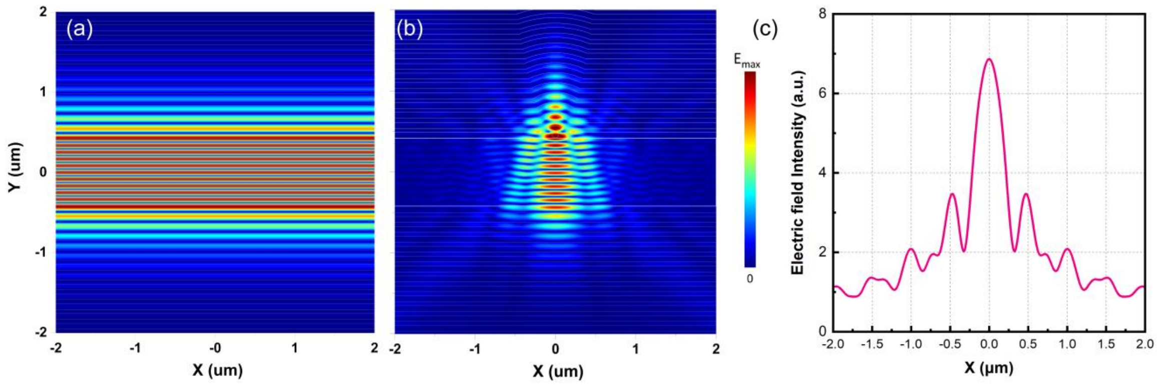

Figure S3. Simulated light field distribution in (**a**) planar and (**b**) curved VCSELs, (**c**) Light field distribution extracted along y = 0 in curved VCSELs.

4. Q-Factor Estimation

For the spectrometer system, the relationship between the measured linewidth Wm, the true linewidth Wt, and the instrument resolution R is given by:

$$W_m \approx \sqrt{W_t^2 + R^2}$$

The spectrometer resolution used in this study is 0.04 nm, it can be estimated that when the measured half-width is 0.15 nm, the measurement error is approximately 4%, and the actual Q-factor is ~0.145 nm.

5. Lasing Behavior of a Coupled Curved VCSELs Pair

As shown in Figure S4, due to the random distribution of the SiO_2 spheres, we identified a curved DBR pair and performed optical pumping at different positions. We find that once the pumping spot aligns with the central region of the coupled curved VCSEL pair [see Figure S4 (b)], the near-field pattern splits into 2 spots, each localized above a DBR. The emission spectrum in Figure S4 (d) shows two peaks at 418.8 nm and 418.4 nm. When the pump source was placed above a specific curved DBR, the near-field pattern degenerated into a single spot [see Figure S4 (c)], appearing spectrally as a monochromatic peak at 418.54 nm. Since the shape of the near-field spot closely matches that of the bottom curved DBR, we confirm that the curved DBR indeed achieves good optical confinement and contributes to improved device performance.

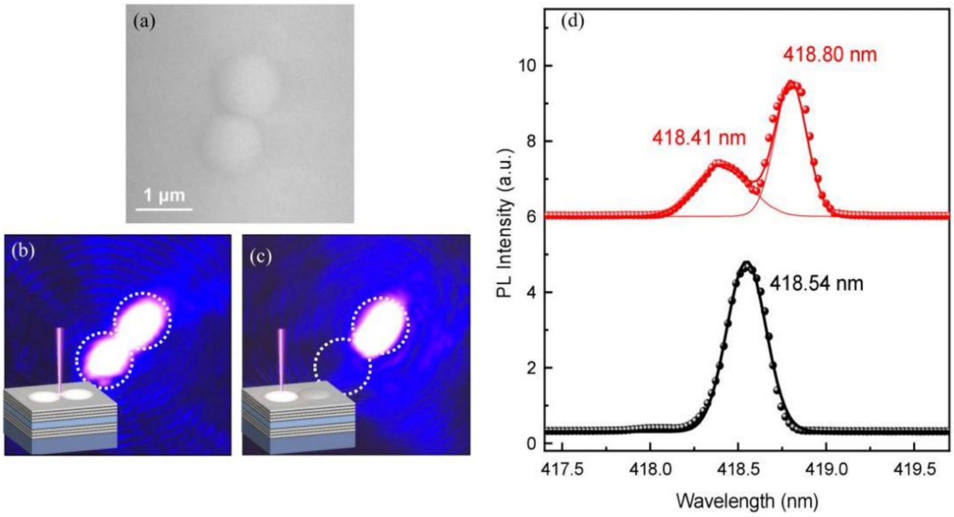

Figure S4. (**a**) SEM image of a coupled curved VCSELs pair. (**b**) Near-field patterns of the coupled VCSELs when pump spot is aligned with the central region and (**c**) one of the curved DBRs. (**d**) Emission spectra of the coupled VCSELs in two pumping configurations.

6. The Recent Reports on GaN-Based VCSELs

The quality factors of recent GaN-based vertical-cavity devices were summarized in Table R1. The Q factor of early GaN vertical-cavity devices were mostly below 1000, whereas those of GaN-based vertical cavities fabricated in recent years range from approximately 1000 to 3000 [32,33,42,43]. Ref. [30] presents a buried micropillar structure similar to the scheme in this paper, with a theoretical Q-factor of ~2000. Notably, the vertical cavity Q-factors obtained by Zhang Baoping’s group typically range from 2000 to 3000, owing to their atomically flat interfaces and shorter cavity lengths. In summary, it can be concluded that the Q-factor of 2800 achieved by the novel microcavity structure employed in this paper is at a favorable level.

Table S1. recent Q-factor reports on GaN-based VCSEL.

Year	Author/Group	Q factor
2023	T. Taniguchi et. al	3400
2014	Zhang Baoping et. al	3570
2014	H. Morkoc's Team (VCU)	1300
2006	J.T. Chu et al. (NCTU, Taiwan)	518
